# Supplementary material for: Secreted LysM proteins are required for niche competition and full virulence in Pseudomonas savastanoi during host plant infection
Source: PLoS Pathog. 2025 Aug 1;21(8):e1013121. doi: 10.1371/journal.ppat.1013121 (PMC12327690; doi:10.1371/journal.ppat.1013121)
Supplement: S2 Table — (PDF) [file ppat.1013121.s002.pdf]

S2 Table. Bacterial strains and plasmids used in this work

Strain	Main features	Reference or source
Escherichia coli		
DH5α	F -, ϕ80d *lacZ*ΔM15, (*lacZYA-argF*)*U169*, *deoR*, *recA1*, *endA*, *hsdR17* (rk - mk -), *phoA*, *supE44*, *thi-1*, *gyrA96*, *relA1*	(98)
BL21 (DE3)	F -, *ompT*, *hsdSB* (rB– mB–), *gal*, *dcm* (DE3).	(99)
***Pseudomonas savastanoi* pv. savastanoi**		
NCPPB 3335	Wild-type strain isolated from olive	(100)
Psv-ΔhrpA	Derived from Psv NCPPB 3335, the complete *hrpA* was deleted and replaced with a Km-resistance gene (KmR)	(37)
Psv-ΔhrpL	Derived from Psv NCPPB 3335, the complete *hrpL* was deleted and replaced with a Km-resistance gene (KmR)	(38)
Δ*lysM1*	Derived from Psv NCPPB 3335, the complete *lysM1* was deleted and replaced with a Km-resistance gene (KmR)	This work
Δ*lysM2*	Derived from Psv NCPPB 3335, the complete *lysM2* was deleted and replaced with a Km-resistance gene (KmR)	This work
Δ*lysM3*	Derived from Psv NCPPB 3335, the complete *lysM3* was deleted and replaced with a Km-resistance gene (KmR)	This work
Δ*lysM4*	Derived from Psv NCPPB 3335, the complete *lysM4* was deleted and replaced with a Km-resistance gene (KmR)	This work
Δ*lysM5*	Derived from Psv NCPPB 3335, the complete *lysM5* was deleted and replaced with a Km-resistance gene (KmR)	This work
Δ*lysM1*::*lysM1*	*lysM1* mutant complemented with gene *lysM1* using pBBR::*lysM1*	This work
Δ*lysM2*::*lysM2*	*lysM2* mutant complemented with gene *lysM2* using pBBR::*lysM2*	This work
Δ*lysM3*::*lysM3*	*lysM3* mutant complemented with gene *lysM3* using pBBR::*lysM3*	This work
Δ*lysM4*::*lysM4*	*lysM4* mutant complemented with gene *lysM4* using pBBR::*lysM4*	This work
Δ*lysM5*::*lysM5*	*lysM5* mutant complemented with gene *lysM5* using pBBR::*lysM5*	This work
Pantoea agglomerans		
DAPP-PG 734	Wild-type strain, isolated from olive	(73)
Erwinia toletana		
DAPP-PG 735	Wild-type strain, isolated from olive	(73)
Bacillus subtilis		
3610	Wild-type strain	(101)

Plasmid	Description	Reference

pGEM-T	Cloning vector containing ori f1 and lacZ (ApR)	(Promega, USA)
plysM1-Km	pGEM-T derivate, contains approximately 1,2 Kb on each side of the *P. savastanoi* pv. savastanoi NCPPB 3335 *lysM1* gene (ApR, KmR)	This work
plysM2-Km	pGEM-T derivate, contains approximately 1,2 Kb on each side of the *P. savastanoi* pv. savastanoi NCPPB 3335 *lysM2* gene (ApR, KmR)	This work
plysM3-Km	pGEM-T derivate, contains approximately 1,2 Kb on each side of the *P. savastanoi* pv. savastanoi NCPPB 3335 *lysM3* gene (ApR, KmR)	This work
plysM4-Km	pGEM-T derivate, contains approximately 1,2 Kb on each side of the *P. savastanoi* pv. savastanoi NCPPB 3335 *lysM4* gene (ApR, KmR)	This work
plysM5-Km	pGEM-T derivate, contains approximately 1,2 Kb on each side of the *P. savastanoi* pv. savastanoi NCPPB 3335 *lysM5* gene (ApR, KmR)	This work
pBBR::lysM1	pBBR1MCS-5 derivative, contains *P. savastanoi* pv. savastanoi NCPPB 3335 open reading frame of the *lysM1* gene and its promoter region	This work
pBBR::lysM2	pBBR1MCS-5 derivative, contains *P. savastanoi* pv. savastanoi NCPPB 3335 open reading frame of the *lysM2* gene and its promoter region	This work
pBBR::lysM3	pBBR1MCS-5 derivative, contains *P. savastanoi* pv. savastanoi NCPPB 3335 open reading frame of the *lysM3* gene and its promoter region	This work
pBBR::lysM4	pBBR1MCS-5 derivative, contains *P. savastanoi* pv. savastanoi NCPPB 3335 open reading frame of the *lysM4* gene and its promoter region	This work
pBBR::lysM5	pBBR1MCS-5 derivative, contains *P. savastanoi* pv. savastanoi NCPPB 3335 open reading frame of the *lysM5* gene and its promoter region	This work
pET28a	A expression vector designed for protein production in *E. coli* under the control of the T7 promoter (KmR)	(Novagen, USA)
pET28a::lysM3	pET28a derivative, contains *P. savastanoi* pv. savastanoi NCPPB 3335 open reading frame of the *lysM3* gene	This work
pET28a::lysM4	pET28a derivative, contains *P. savastanoi* pv. savastanoi NCPPB 3335 open reading frame of the *lysM4* gene	This work
