# Supplementary material for: Secreted LysM proteins are required for niche competition and full virulence in Pseudomonas savastanoi during host plant infection
Source: PLoS Pathog. 2025 Aug 1;21(8):e1013121. doi: 10.1371/journal.ppat.1013121 (PMC12327690; doi:10.1371/journal.ppat.1013121)
Supplement: S3 Table — (PDF) [file ppat.1013121.s003.pdf]

S3 Table. Oligonucleotides used in this work

Purpose	Name	Sequence (5´-3´)
**Mutagenize**		
lysM1	F-Up-lysM1	ACAAACCCTGATACTCAGGAGC
	R-Up-lysM1	CCCTATAGTGAGTCGAATTCAGCCCATCCCTTGTTTAAAGATTCC
	F-Dw-lysM1	GAATTCGACTCACTATAGGGTCCCCCTAAGGCAACAACC
	R-Dw-lysM1	TTCATCAGGTGTTTCATCGCC
lysM2	F-Up-lysM2	AGACATCGACCTTGAAGGCTATGG
	R-Up-lysM2	CCCTATAGTGAGTCGAATTCGCGAACCCTTATAGAAACCACAG
	F-Dw-lysM2	GAATTCGACTCACTATAGGGACCCGTTGTTTTCTTGAGCAGG
	R-Dw-lysM2	CTTCTTCGCTGAGCATGGCC
lysM3	F-Up-lysM3	GTGCCGGCTACTGGATTGC
	R-Up-lysM3	CCCTATAGTGAGTCGAATTCCCGCCACTCCCTTTACTGG
	F-Dw-lysM3	GAATTCGACTCACTATAGGGTAGTTGCCAGCCTGTTCCG
	R-Dw-lysM3	AAGGATCTCACGCAGGCG
lysM4	F-UP-lysM4	AGACGCGGAATATCCAGCACCA
	R-UP-lysM4	CCCTATAGTGAGTCGGATCCGCGGTGAATCCCTTTATCATGT
	F-DW-lysM4	GGATCCGACTCACTATAGGGAGGTATGTTTCAGTTCTGCTAC
	R-DW-lysM4	TGTGCTCAACGGTCTCGACCAG
lysM5	F-UP-lysM5	AGTTTGCTGTCGGCTGCC
	R-UP-lysM5	CCCTATAGTGAGTCGGATCCACCTAACCCCATATCTATTTGA
	F-DW-lysM5	GGATCCGACTCACTATAGGGCCGATCTTCTGCTCGACGGCAC
	R-DW-lysM5	CGCCCGTCGCGGAAGCGTACTT
**Complement**		
lysM1	F-RBS-lysM1	TTAAATGGTACCGGCAACAATTTGAATAATT
	R- lysM1	GGTGGTGGATCCTCAGGCCAGCCGCGAAAGC
lysM2	F-RBS-lysM2	TTAAATGGTACCCCTGAGACGAAGCCGGGCG
	R-lysM2	GGTGGTGGATCCTCAGCTGATGCCCGCCAAC
lysM3	F-RBS-lysM3	TTAAATGGTACCTTTAGTCCTTTGGTGAGTT
	R-lysM3	TTAAATGAATTCTGCTCCAGCACGCCTTCAG
lysM4	F-RBS-lysM4	TTAAATGGTACCTAAAGTCAATGTTGGGTGC
	R-lysM4	TTAAATGGATCCTTAAGGATTTCGAACCTTGTCCAT
lysM5	F-RBS-lysM5	TTAAATGGTACCACGGAGAAGGTCGGTCCGT
	R-lysM5	TTAAATGGATCCTCATTGCTGAGTCGCCACCTCGGAA
**Overexpress**		
lysM3	F-lysM3	GGTGGTCATATGTGCTCCAGCACGCCTTCAG
	R-lysM3	TTAAATGAATTCTGCTCCAGCACGCCTTCAG
lysM4	F-lysM4	TTAAATCATATGGTGCAACTCAGGGAAGGCTATCCGC
	R-lysM4	TTAAATGGATCCTTAAGGATTTCGAACCTTGTCCAT
