# Supplementary material for: Secreted LysM proteins are required for niche competition and full virulence in Pseudomonas savastanoi during host plant infection
Source: PLoS Pathog. 2025 Aug 1;21(8):e1013121. doi: 10.1371/journal.ppat.1013121 (PMC12327690; doi:10.1371/journal.ppat.1013121)
Supplement: S1 Fig — The secretome was obtained after 12 hours of incubation in HIM medium. The analysis highlights significantly enriched gene ontology (GO) functional categories, classified by molecular function and cellular localization. The X-axis represents the number of proteins associated with each category, while the color indicates the degree of enrichment (fold enrichment). Fold enrichment represents the frequency of a given GO term in the secretome relative to its frequency in the complete deduced proteome of strain NCPPB 3335. The analysis was performed with a false discovery rate (FDR) cut-off of 0.05. (PDF) [file ppat.1013121.s004.pdf]

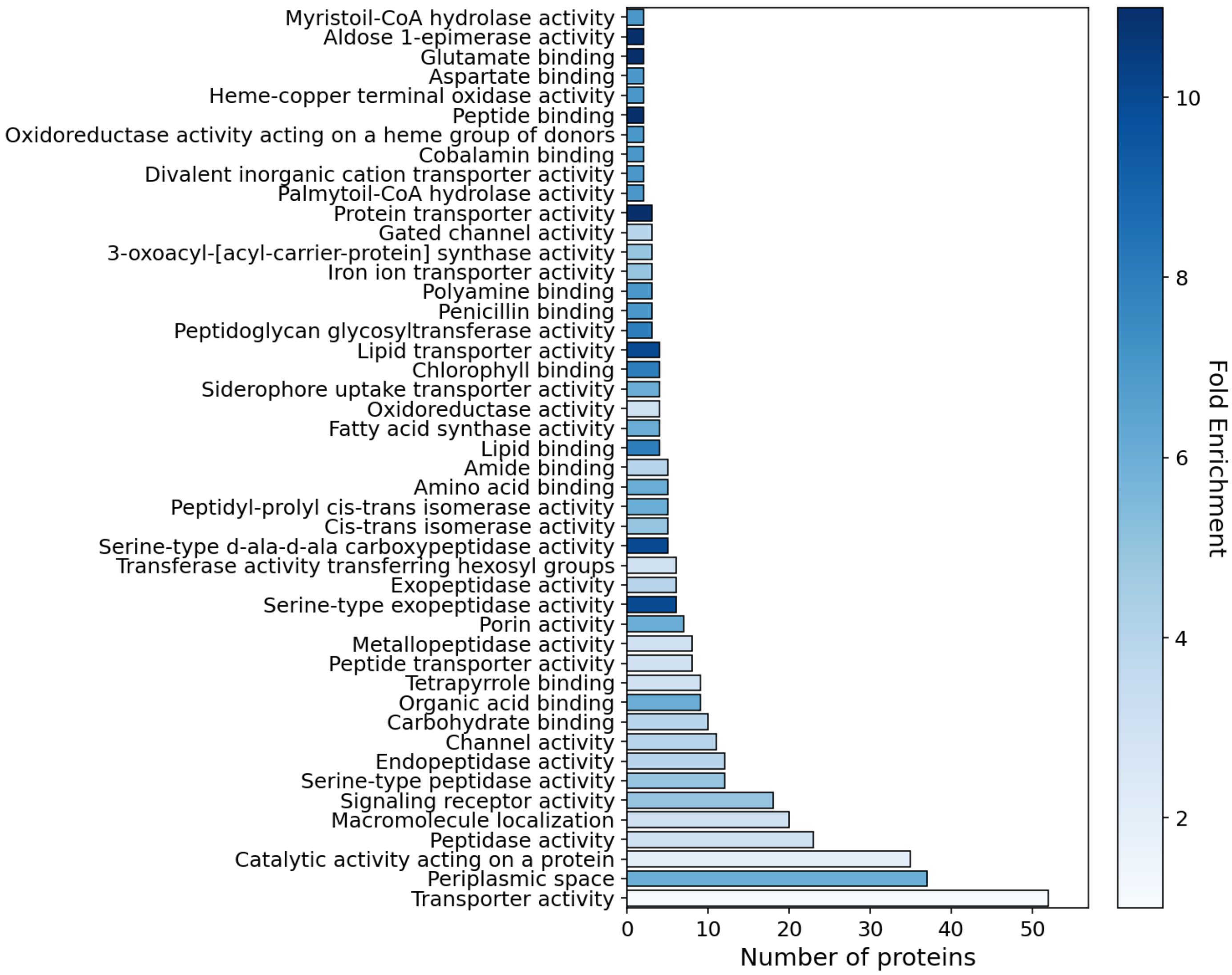

S1 Figure. Functional enrichment analysis of the *Pseudomonas savastanoi* NCPPB 3335 secretome performed using ShinyGO. The secretome was obtained after 12 hours of incubation in HIM medium. The analysis highlights significantly enriched gene ontology (GO) functional categories, classified by molecular function and cellular localization. The X-axis represents the number of proteins associated with each category, while the color indicates the degree of enrichment (fold enrichment). Fold enrichment represents the frequency of a given GO term in the secretome relative to its frequency in the complete deduced proteome of strain NCPPB 3335. The analysis was performed with a false discovery rate (FDR) cut-off of 0.05.
