# Supplementary material for: Secreted LysM proteins are required for niche competition and full virulence in Pseudomonas savastanoi during host plant infection
Source: PLoS Pathog. 2025 Aug 1;21(8):e1013121. doi: 10.1371/journal.ppat.1013121 (PMC12327690; doi:10.1371/journal.ppat.1013121)
Supplement: S2 Fig — (A) Growth profiles of wild-type P. savastanoi pv. savastanoi NCPPB 3335 and its ΔlysM mutants in rich medium (KB). The graph represents the average of three independent experiments. (B) Colony forming units (CFU)/mL of bacterial populations after 24 hours of growth in KB. Bars represent the mean CFU/mL ± standard error from three biological replicates. Letters above the bars denote statistically significant differences (p < 0.05) based on ANOVA followed by Tukey’s t-test. (PDF) [file ppat.1013121.s005.pdf]

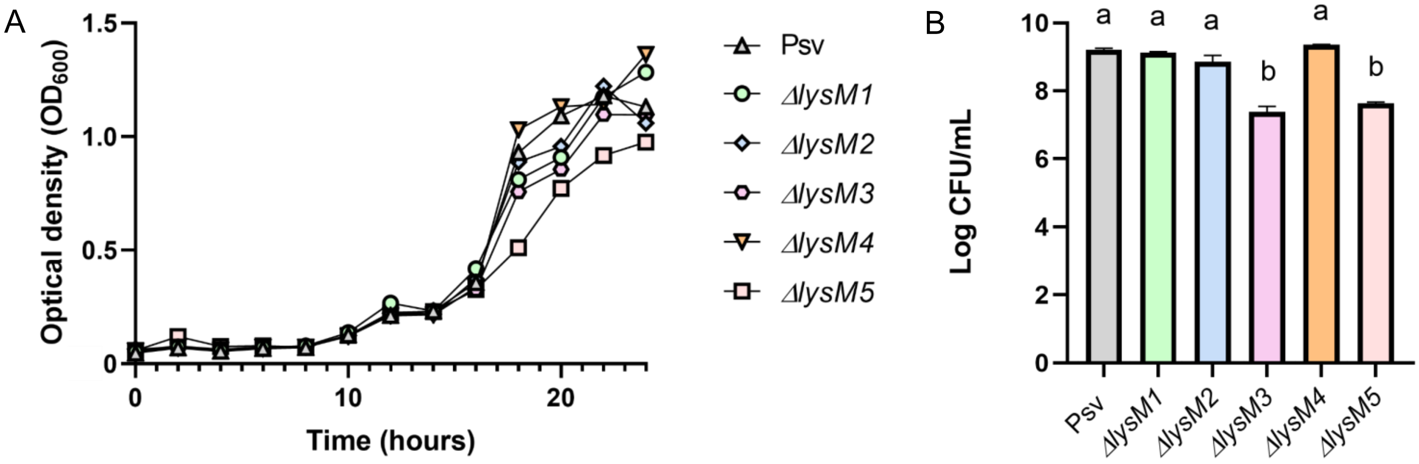

S2 Figure. Absence of LysM3 and LysM5 in *P. savastanoi* pv. savastanoi NCPPB 3335 reduces maximum cell counts during growth in culture medium. (A) Growth profiles of wild-type *P. savastanoi* pv. savastanoi NCPPB 3335 and its Δ*lysM* mutants in rich medium (KB). The graph represents the average of three independent experiments. (B) Colony forming units (CFU)/mL of bacterial populations after 24 hours of growth in KB. Bars represent the mean CFU/mL ± standard error from three biological replicates. Letters above the bars denote statistically significant differences ($p < 0.05$) based on ANOVA followed by Tukey's t-test.
