# Supplementary material for: Secreted LysM proteins are required for niche competition and full virulence in Pseudomonas savastanoi during host plant infection
Source: PLoS Pathog. 2025 Aug 1;21(8):e1013121. doi: 10.1371/journal.ppat.1013121 (PMC12327690; doi:10.1371/journal.ppat.1013121)
Supplement: S3 Fig — Crucial conserved amino acids are marked with a red arrow. (A) Structural superimposition of Lys3-LysM (AlphaFold model) and P60-LysM (PDB entry 4zu3) domains showing fold conservation (RMSD: 1.24 Å). (B) Sequence alignment of the LysM domains of P60 and LysM3, with color intensity indicating conservation levels and bars showing conservation scores. Alignment quality, consensus sequence, and occupancy are also displayed. (C) Superimposition of LysM3-M23 Alphafold model and Csd1-M23 crystal coordinates (PDB entry 5j1l) showing structural conservation (RMSD: 1.17 Å). (D) Sequence alignment of the M23 domains of Csd1 and LysM3, following the same annotation scheme as in panel B. (E) Structural superimposition of LysM3-M23 (AlphaFold model) and Pgp3-M23 crystal coordinates (PDB entry 6jn1) showing structural conservation (RMSD: 0.83 Å). (F) Sequence alignment of the M23 domains of Pgp3 and LysM3, following the same annotation scheme as in panel B. (PDF) [file ppat.1013121.s006.pdf]

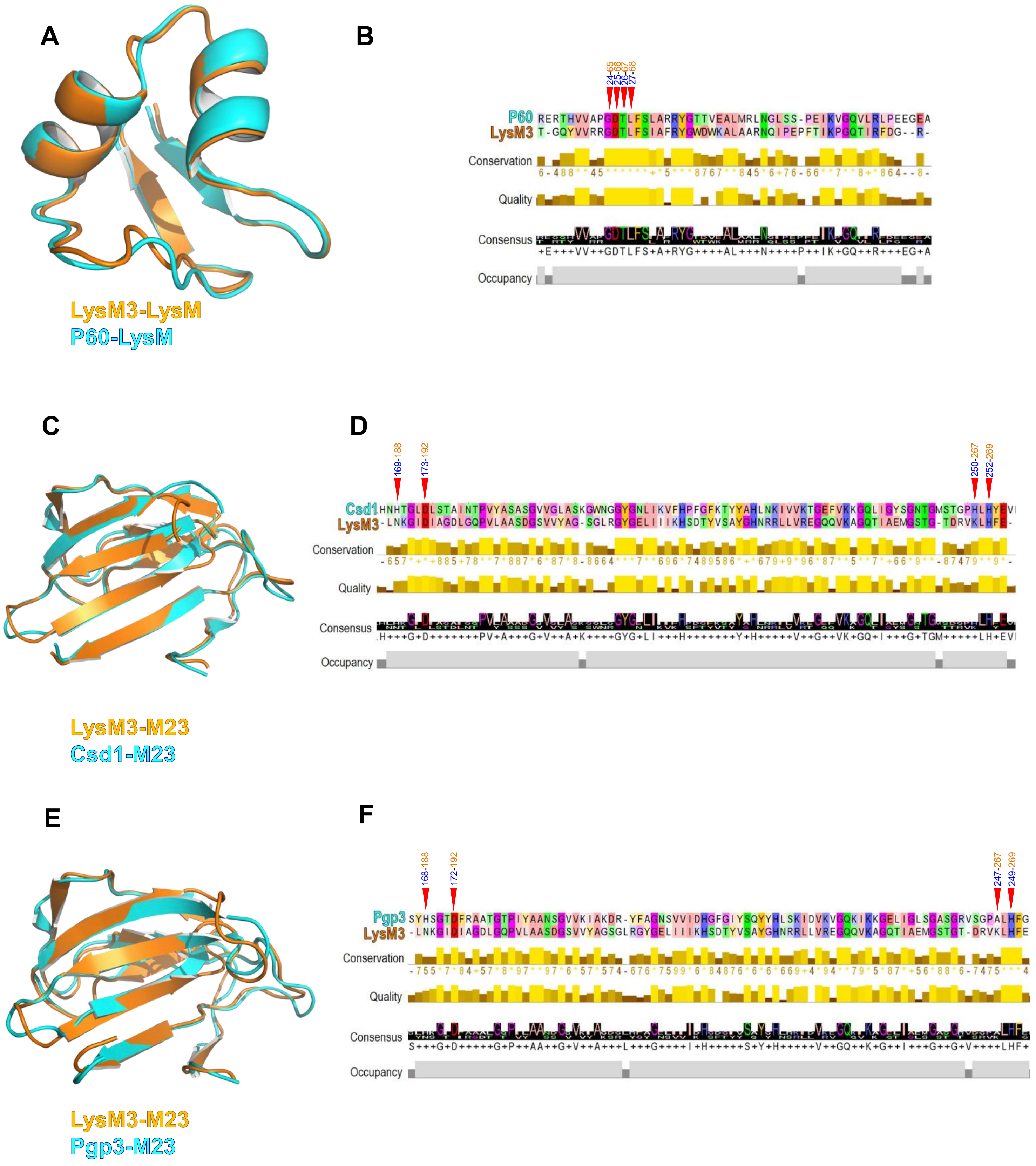

S3 Figure. Structural and sequence comparison of the LysM and M23 domains of LysM3 with other proteins. Crucial conserved amino acids are marked with a red arrow. (A) Structural superimposition of Lys3-LysM (AlphaFold model) and P60-LysM (PDB entry 4zu3) domains showing fold conservation (RMSD: 1.24 Å). (B) Sequence alignment of the LysM domains of P60 and LysM3, with color intensity indicating conservation levels and bars showing conservation scores. Alignment quality, consensus sequence, and occupancy are also displayed. (C) Superimposition of LysM3-M23 Alphafold model and Csd1-M23 crystal coordinates (PDB entry 5j1l) showing structural conservation (RMSD: 1.17 Å). (D) Sequence alignment of the M23 domains of Csd1 and LysM3, following the same annotation scheme as in panel B. (E) Structural superimposition of LysM3-M23 (AlphaFold model) and Pgp3-M23 crystal coordinates (PDB entry 6jn1) showing structural conservation (RMSD: 0.83 Å). (F) Sequence alignment of the M23 domains of Pgp3 and LysM3, following the same annotation scheme as in panel B.
